# Cryptococcal Meningoencephalitis With Herpes Zoster Coinfection in a Retrovirus-Positive Adolescent Male With a Normal CD4 Count: A Rare Case

**DOI:** 10.7759/cureus.82271

**Published:** 2025-04-14

**Authors:** Mrunalini kulkarni, Unmesh R Sawant, Nidhisha Kakarla

**Affiliations:** 1 Pediatrics and Neonatology, Bharati Vidyapeeth (Deemed to be University) Medical College and Hospital, Sangli, IND; 2 Pediatrics and Child Health, Bharati Vidyapeeth (Deemed to be University) Medical College and Hospital, Sangli, IND

**Keywords:** cryptococcal meningitis, cryptococcal meningoencephalitis, cryptococcus gatti, cryptococcus neoformans, herpes zoster, hiv aids, hiv/aids

## Abstract

Cryptococcal meningoencephalitis is a grave and lethal form of an opportunistic infection usually occurring in immunocompromised patients (i.e., those with AIDS, organ transplant recipients, or those on immunosuppressive therapy) caused by *Cryptococcus* species, namely *Cryptococcus neoformans,* which has a worldwide distribution, or *Cryptococcus gattii,* which is predominantly found in tropical, subtropical, and temperate regions. *Cryptococcus* species are usually found in soil contaminated with bird or animal droppings in the form of cryptococcal spores. The former occurs predominantly in immunocompromised patients, affecting the central nervous system (CNS), while the latter occurs in immunocompetent patients, affecting the pulmonary system predominantly. Transmission occurs through these spores, which subsequently disseminate to the CNS through the hematogenous route. Manifestations include fever, headache, nausea, altered mental status, and meningismus in certain cases, potentially advancing to neurological problems such as elevated intracranial pressure, blindness, and seizures. Presentation in immunocompetent individuals is indolent, so diagnosis and treatment are delayed. Timely diagnosis and immediate treatment are essential to avert complications and mortality. Over the past few years, cryptococcal infection has seen an upward trend globally. Despite the availability of antifungal therapy, it has emerged as a significant public health concern, rendering it a therapeutically challenging case. Moreover, infrequent coinfections with bacteria or viruses might complicate therapy and prognosis. We hereby report a rare case of cryptococcal meningoencephalitis with herpes zoster coinfection occurring in an HIV patient with a normal CD4 count.

## Introduction

Neuromeningeal cryptococcosis is an opportunistic infection caused by a ubiquitous environmental encapsulated yeast (*Cryptococcus* species), usually seen in patients with HIV infection and advanced immunodeficiency (CD4 count <100 cells/μl) [1]. It induces inflammation of the meninges encasing the spinal cord and brain, and occasionally, brain tissue may also be affected. Cryptococcal meningitis is an opportunistic infection and an AIDS-defining illness, with an estimated global incidence of 220,000 cases per year [2,3]. A review of the global HIV/AIDS data from 2014 showed that cryptococcal meningoencephalitis was responsible for 181,100 deaths and 15% of AIDS-related deaths [4-6]. High mortality rates were observed in resource-constrained nations, attributed to diagnostic delays and insufficient treatment, compounded by the presence of coinfections, which significantly worsened prognosis [7,8].

Cryptococci usually cause severe disease in the immunocompromised but can also rarely affect the immunocompetent patients. The notable element of our case is the rare occurrence of cryptococcal meningoencephalitis and herpes zoster coinfection in an HIV patient with a normal CD4 count. Given the altered sensorium and history of convulsions, cerebrospinal fluid (CSF) examination and India ink staining were performed, yielding a positive result for *Cryptococcus*. Accordingly, antifungals were started, and the patient had a favorable outcome. Despite modern therapies, cryptococcal meningitis remains a significant health burden, further exacerbated by coinfections, resulting in increased mortality and morbidity. Hence, the purpose of presenting this rare case is to emphasize the significance of prompt clinical suspicion and interdisciplinary intervention, which resulted in a favorable clinical outcome in our case.

## Case presentation

A 17-year-old male patient with a medical history of HIV/AIDS (on antiretroviral therapy (ART), current CD4 count 451 cells/mm³ and viral load 14788 copies/mm³) presented to the emergency department following a seizure episode characterized by repetitive movements of the upper and lower limbs with rigidity lasting 20 minutes, occurring two hours prior to admission, accompanied by frothing at the mouth, upward eye rolling, altered consciousness, and aggressive behavior.

There was no prior occurrence of fever, cough, cold, ear discharge, head trauma, tongue laceration, vomiting, involuntary passage of urine and stools, loose stools, or any abnormal movements. There was no history of systemic diseases (thyroid disorders, tuberculosis, cardiovascular disease, renal disease), no history of cancer, and no prior similar complaints. All family members are retrovirus-positive and are on ART.

**Figure 1:**
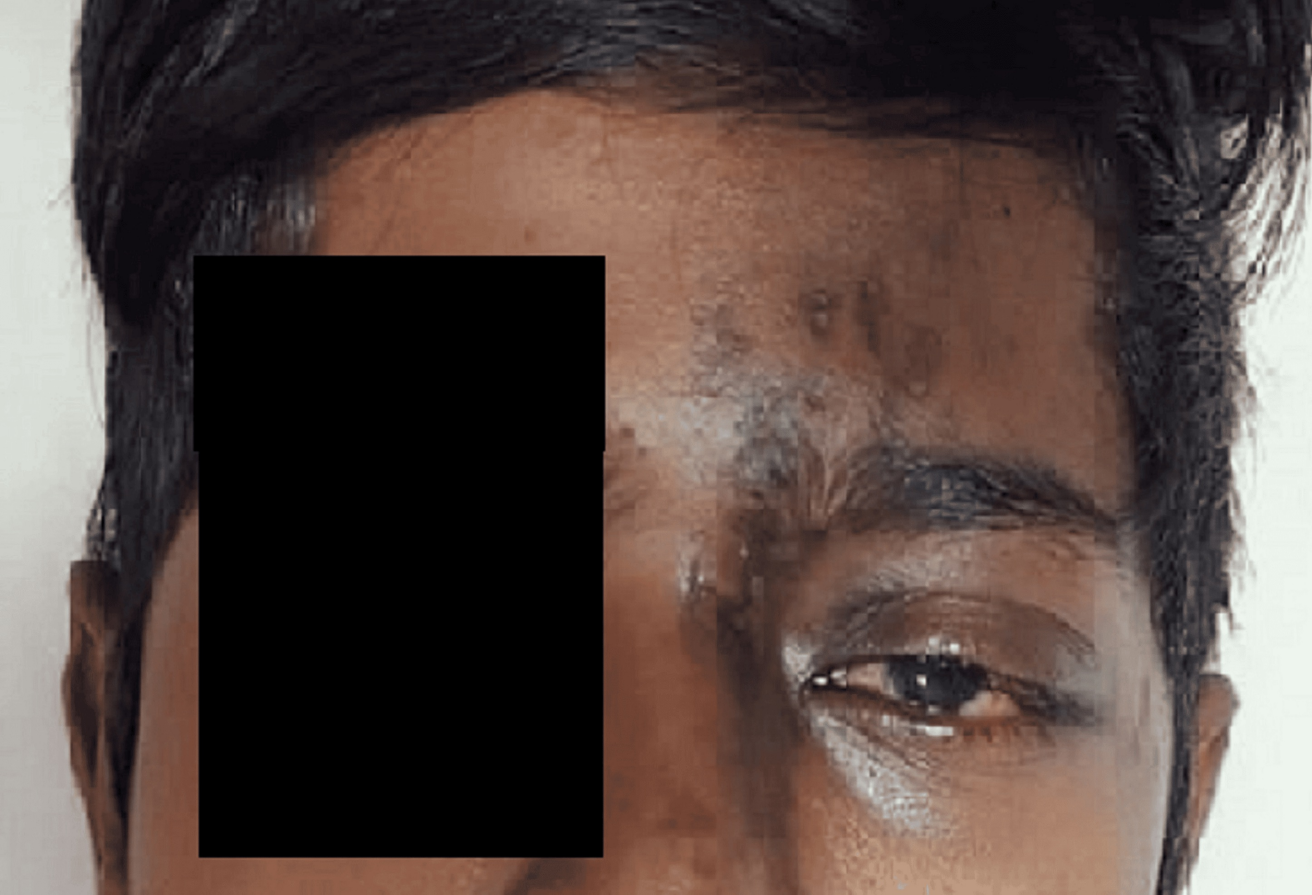
Patient showing ptosis of the left eyelid and herpetic lesions The patient consented, and a written, signed consent statement was provided to the journal, granting permission to have their identity revealed in an open access publication.

Upon examination, the patient was hemodynamically stable (blood pressure: 120/80 mmHg, heart rate: 90 bpm), exhibited violent behavior, and was making incomprehensible sounds. On general examination, multiple pinheaded vesicles were seen on the left frontal and infraorbital regions. There was conjunctival congestion and lacrimation of the left eye. His Glasgow Coma Scale score was 11/15. The ocular examination revealed a dilated left pupil that was not reactive to light and a noted ptosis of the left eyelid (Figure 1). The right pupil was normal and responsive to light. Ophthalmology referral was advised, following which the patient was found to have postherpetic left third cranial nerve palsy. Other cranial nerves were normal, tone and power were normal, and no neck stiffness was found. On referral to the dermatologist, a diagnosis of herpes zoster was made, and tablet acyclovir 400 mg was given. The rest of the examination was unremarkable.

The provisional diagnosis was acute meningoencephalitis in a retroviral disease-positive patient. Several laboratory tests were done to rule out causative organisms, including CSF examination, blood analysis, coagulation profile, renal function assessment, and MRI as the imaging modality.

**Figure 2:**
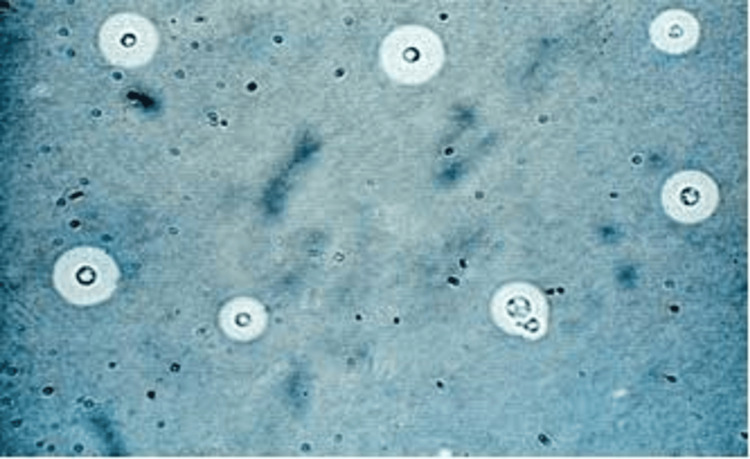
India ink staining showing Cryptococcus at 10X magnification

The CSF analysis revealed lymphocytosis, elevated protein content, and increased glucose levels (Table 1). The India ink staining of the CSF sample (Figure 2) showed a few capsulated budding yeast cells, proving *Cryptococcus*. A bacteriology report revealed a few scanty pus cells with occasional gram-positive budding yeast cells. Ziel-Nelson staining for acid-fast bacilli was negative.

**Table 1:** CSF analysis after lumbar puncture CSF: cerebrospinal fluid, RBC: red blood cells, WBC: white blood cells

CSF examination	Result	Reference range
Specific gravity	1.020	1.020
Color	Colorless	Colorless
Appearance	Clear	Clear
RBC	00	≤0 mm^3^
WBC	03	0-5 mm^3^
Lymphocytes	100	20-40 cells/ HPF
Protein	85.7	15-45 mg/dL
Sugar	79	50-75 mg/dL

A complete blood count examination revealed normal levels of hemoglobin and normal levels of WBC, polymorphs, lymphocytes, and platelet count. The coagulation profile revealed normal prothrombin time, activated partial thromboplastin time, and the international normalized ratio. Renal function tests revealed normal blood urea and serum creatinine with normal levels of electrolytes (Table 2).

**Table 2:** Admission laboratory values and electrolyte values compared to normal ranges WBC: white blood cells

Laboratory test	Result	Reference range
Hemoglobin	13.7	15-19 g/dl
WBC count	12.28 × 10^3^	3.4-10.8 × 10^3^/mm3
Platelets	1.97 × 10^3^	1.5-4.5 × 10^3^/μL of blood
Serum sodium	139	134-144 mEq/L
Serum potassium	3.7	3.5-5.2 mEq/L
Serum calcium	9.8	8.4-10.5 mEq/L
Serum ionic calcium	1.1	0.9-1.4 mEq/L
Serum magnesium	1.8	1.9-2.4 mEq/L
Serum phosphorus	3.5	3.5-5.5 mEq/L
Lactic acid	35.7	0.5-2.2 mmol/L
Blood urea	21	7-23 mg/dL
Serum creatinine	0.5	0.60-1.30 mg/dL

**Figure 3:**
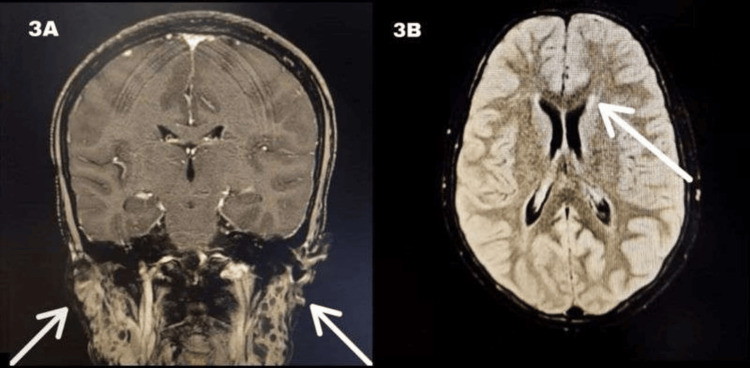
(A) bilateral parotid enlargement and (B) hyperdense foci on post-contrast MRI These image findings are indicative of benign lymphoepithelial lesions, a salivary gland disease seen commonly in HIV positive individuals. MRI: magnetic resonance imaging, HIV: human immunodeficiency virus

**Figure 4:**
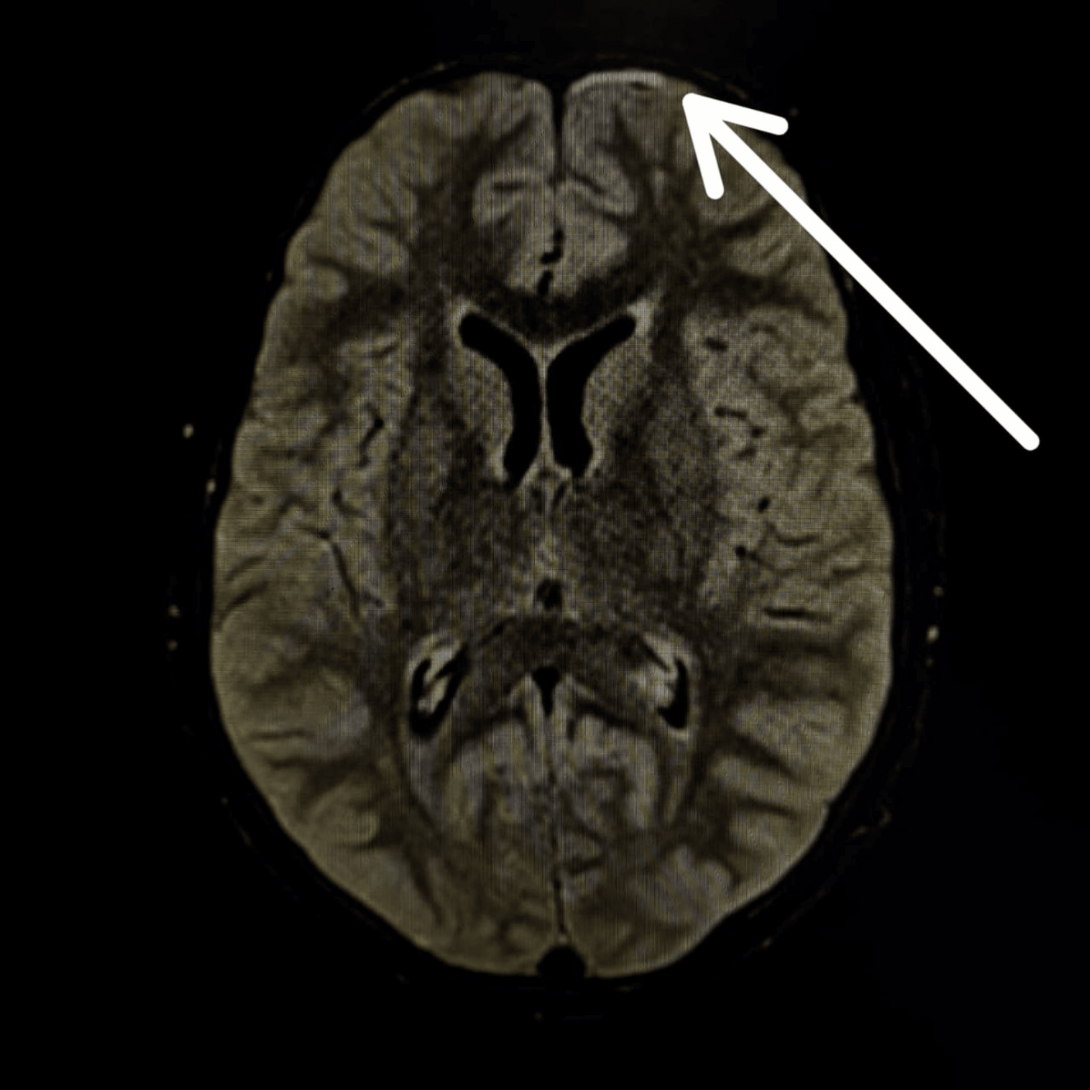
Leptomeningeal enhancement on FLAIR MRI FLAIR MRI: fluid attenuated inversion recovery magnetic resonance imaging

An MRI of the brain with contrast suggested likely infectious foci, a small mucous retention cyst in the left maxillary sinus, and enlarged bilateral parotids, with multiple small hyperintense foci classic for lymphoepithelial cysts in the parotid glands (Figure 3A) with multiple areas of altered signal intensity (ASI). The post-contrast study showed enhancement, suggesting microabscesses/infiltrates with dilated Virchow-Robin spaces (Figure 3B). The frontal white matter also displayed multiple areas of ASI. Additionally, leptomeningeal enhancement on fluid attenuated inversion recovery (FLAIR) was seen (Figure 4).

Hence, a diagnosis of cryptococcal meningoencephalitis with left-sided postherpetic third cranial nerve palsy was confirmed. Upon admission, the patient was administered intravenous levetiracetam and a fixed-dose combination tablet of tenofovir 300 mg, dolutegravir 50 mg, and lamivudine 300 mg for seizures. After giving the injection of mannitol, his mental status improved. Subsequent to the ophthalmology and dermatology consultation, acyclovir tablets 400 mg every eight hours, acyclovir ointment, moxifloxacin eye drops, and Refresh tear drops were started. Later, after the CSF examination revealed cryptococcal infection, the patient commenced induction therapy with liposomal amphotericin B at a dosage of 10 mg/kg daily for 14 days, in conjunction with fluconazole 400 mg administered twice daily for the same duration. Flucytosine was not administered due to financial constraints. During the consolidation phase, fluconazole was prescribed at a dosage of 200 mg every 12 hours for a duration of two months. The patient exhibited improvement with these medications; hence, a repeat of laboratory tests was recommended. The CSF investigation showed no pus cells, the bacteriology report indicated no organisms, and Ziel-Nelson staining was negative for acid-fast bacilli and India ink. The ink preparation of the CSF specimen tested negative for *Cryptococcus*.

Subsequent to a positive result, the patient is undergoing maintenance therapy with fluconazole 400 mg administered twice daily for one year, alongside ART, anticonvulsants, and antiviral drugs for herpes. The patient's condition stabilized two months post-hospitalization, leading to his discharge. The patient received counseling regarding adherence to ART and was instructed on monthly follow-up appointments.

## Discussion

Over the past two decades, central nervous system (CNS) cryptococcosis has emerged as a major opportunistic infection in the immunocompromised population. Hence, spreading awareness about the disease, understanding prevention and control measures, and adhering to treatment protocols are essential. Cryptococcal illness is primarily attributed to *Cryptococcus neoformans*, which exhibits a worldwide distribution, with large numbers reported in the far East, and *Cryptococcus gattii*, which is more predominant in tropical, subtropical, and temperate regions, including India, Australia, Africa, the United States, Canada, Australasia, and South America [9-11]. Cryptococcosis is typically linked to HIV infection or other forms of immunosuppression, including solid organ transplant recipients and individuals on systemic immunosuppressive treatments such as glucocorticoids, chemotherapy, or disease-modifying drugs. Epidemiology and pathogenesis are explained in Table 3.

**Table 3:** Epidemiology and pathogenesis of cryptococcosis according to infecting species CNS: central nervous system, HIV: human immunodeficiency virus

Species	Cryptococcus neoformans	*Cryptococcus gattii* species complex
Geographic distribution	Worldwide	Predominantly in tropical, subtropical, and temperate regions (India, Australia, South America, Africa, the United States, and Canada)
Ecology	Avian guano, bark, tree trunk hollows	Tropical and temperate rainforest, especially eucalyptus trees, rotting wood, and soil
Host range	Predominantly immunocompromised patients (especially HIV patients), but can also be seen in immunocompetent patients	Immunocompetent individuals, sporadically in immunosuppressed patients
Transmission	Inhalation of cryptococcal spores followed by hematogenous dissemination	Inhalation of cryptococcal spores followed by hematogenous dissemination
Target organs	CNS	Pulmonary system predominantly

This case shows that cryptococcal meningoencephalitis and herpes zoster can happen together very rarely in an HIV patient with a normal CD4 count. Presentation in immunocompetent individuals is atypical. Our patient presented with the classic features, including fever, headache, and altered mental status; however, meningismus was not seen. The obscurity of certain symptoms and their often insidious expression complicates the diagnosis [12]. Additional prevalent symptoms encompass cervical rigidity, photophobia, disorientation, visual impairments, and auditory deficits [13]. Further complications like increased intracranial pressure can cause blindness, disc edema, seizures, and intractable headaches. Moreover, complications can also emerge because of adverse reactions to antifungal and antiretroviral drugs required for treatment. Our patient did not exhibit any additional symptoms or complications and responded favorably to the treatment.

Imaging studies and laboratory tests facilitate the diagnosis. Lumbar puncture is used for CSF analysis and intracranial pressure management [13]. India ink microscopy serves as the principal diagnostic method for detecting *Cryptococcus* due to its accessibility and cost-efficiency. A CSF fungal culture is regarded as the gold standard for diagnosing cryptococcal meningitis; nonetheless, it may yield false-negative results when the fungal load is minimal. Cryptococcal antigens in serum or CSF media can be diagnostic in asymptomatic patients. Imaging studies include CT scans and MRI scans. CT scans of the brain usually show nonspecific features such as brain atrophy, diffuse edema, and rarely hydrocephalus [13,14]. Brain MRIs in patients with cryptococcal meningitis often show dilated perivascular spaces, leptomeningeal enhancement, pseudocysts, cryptococcoma, and hydrocephalus [13,14]. Abnormal brain imaging is proven to be a poor prognostic factor in cryptococcal meningitis. Despite the presence of risk factors such as abnormal imaging and immunosuppression, the patient's favorable prognosis was attributed to their compliance with treatment and a normal CD4 count.

The treatment of cryptococcal meningitis includes antifungal therapy administered in three phases: induction, consolidation, and maintenance, along with the management of intracranial pressure. It is administered as per the Infectious Disease Society of America's (IDSA) 2010 guidelines or the World Health Organization's (WHO) 2022 guidelines [15]. In our case, we adhered to WHO guidelines (Table 4).

**Table 4:** WHO guideline for cryptococcosis in HIV-infected patients, 2022 All doses refer to children and adolescents, and for adults, refer to the WHO guidelines. ^a^ 10 mg/kg, ^b ^100 mg/kg/day in four divided doses, ^c ^1 mg/kg/day, and ^d ^3–4 mg/kg/day Note: For children and adolescents, up to a maximum of 800 mg of fluconazole Daily is recommended WHO: World Health Organization, HIV: human immunodeficiency virus [15]

Phase	Induction phase week 1	Week 2	Consolidation phase	Maintenance phase
Preferred	Single-dose liposomal amphotericin B^a^ plus flucytosine^b^ combined with fluconazole	-	Fluconazole 6–12 mg/kg per day	Fluconazole 6–12 mg/kg per day
If liposomal amphotericin is unavailable	Amphotericin B deoxycholate^c^ plus flucytosine	Fluconazole 12 mg/kg/ day	Fluconazole 6–12 mg/kg per day	Fluconazole 6-12 mg/kg per day
If no amphotericin formulation is unavailable	Fluconazole 12 mg/kg/ day plus flucytosine^b^	-	Fluconazole 6–12 mg/kg per day	Fluconazole 6–12 mg/kg per day
If flucytosine is unavailable	If liposomal amphotericin B^d^ plus fluconazole 12 mg/kg/day	-	Fluconazole 6–12 mg/kg per day	Fluconazole 6–12 mg/kg per day
If liposomal amphotericin B plus flucytosine is unavailable	Amphotericin B deoxycholate^c^ plus fluconazole 12 mg/kg/day	-	Fluconazole 6–12 mg/kg per day	Fluconazole 6–12 mg/kg per day

Extensive prospective trials have demonstrated fluconazole as the most efficacious treatment; nevertheless, itraconazole at a dosage of 200 mg orally every day for nearly one year serves as an alternative. Access to liposomal amphotericin B and flucytosine has been a global challenge, leading many regions to depend exclusively on fluconazole monotherapy, which is less efficacious. Therefore, improving global access to optimal antifungal medication is very crucial, particularly in the lower- and middle-income countries. Clinical trials found that therapy with a combination of amphotericin B plus flucytosine was superior to amphotericin B alone or fluconazole monotherapy. Similarly, therapy with a combination of fluconazole plus flucytosine seems to be superior to fluconazole alone, although this regimen is more toxic than fluconazole monotherapy. Lipid formulations of amphotericin B in *Cryptococcus* meningitis have some toxicity profile advantage over the conventional amphotericin B formulation when used alone or in combination with flucytosine [16,17].

Management of elevated intracranial pressure in this context may involve conservative measures, including the initial use of osmotic agents such as mannitol, serial lumbar punctures, and temporary percutaneous lumbar drainage or ventriculostomy for patients necessitating recurrent daily drainage. For CSF opening pressures ≥25 cm H₂O with signs of elevated intracranial pressure during induction therapy, CSF drainage should be performed to target a goal CSF pressure of less than 20 cm H₂O [16,17]. If conservative treatment fails, a surgical alternative available includes a ventriculoperitoneal shunt [16,17]. This case underscores the importance of timely clinical suspicion, interdisciplinary action, and adherence to guidelines for favorable outcomes in therapeutically demanding situations. Clinicians must recognize the atypical manifestations of cryptococcal meningitis and refrain from dismissing it in immunocompetent patients.

Despite the presence of poor prognostic factors such as altered mental sensorium, abnormal imaging, and herpes zoster coinfection, our patient exhibited a significant clinical improvement due to prompt management and compliance with treatment. If left untreated, the disease is universally fatal within days to weeks.

## Conclusions

Cryptococcal meningitis is an opportunistic central nervous system illness caused by *Cryptococcus neoformans* or *Cryptococcus gattii*, primarily impacting immunocompromised persons, particularly those with AIDS. An increasing trend of cryptococcal infections and related mortality has been noted globally. Typical manifestations include headaches, nausea, and altered sensorium, potentially leading to severe neurological complications if neglected. Despite advancements in treatment modalities, cryptococcal meningitis is still a significant public health hazard with high morbidity and mortality rates. Nevertheless, rare coinfections with bacteria or viruses such as herpes zoster can complicate the treatment and prognosis. Timely diagnosis through laboratory tests, imaging examinations, and suitable antifungal treatment is essential for managing cryptococcal meningitis and enhancing patient outcomes. Here, we describe a unique case of herpes zoster and cryptococcal coinfection in a patient with AIDS with a normal CD4 count. Clinicians should be aware of the atypical presentation of cryptococcal meningitis and refrain from dismissing it in immunocompetent individuals. Our patient had a favorable outcome despite the presence of poor prognostic factors, considering the compliance with treatment, and is on maintenance therapy presently. The case emphasizes the necessity of enhancing global access to effective antifungal treatment, especially in low-income and middle-income nations, in light of the rising global incidence of cryptococcal meningitis.
